# Global Transcriptional Insights of Pollen-Pistil Interactions Commencing Self-Incompatibility and Fertilization in Tea [*Camellia sinensis* (L.) O. Kuntze]

**DOI:** 10.3390/ijms20030539

**Published:** 2019-01-28

**Authors:** Romit Seth, Abhishek Bhandawat, Rajni Parmar, Pradeep Singh, Sanjay Kumar, Ram Kumar Sharma

**Affiliations:** 1 Biotechnology Department, CSIR-Institute of Himalayan Bioresource Technology, Palampur, Himachal Pradesh 176061, India; romit_seth18@yahoo.com (R.S.); abhishek.bhandawat@gmail.com (A.B.); rajni.parmar03@gmail.com (R.P.); ps111186@gmail.com (P.S.); sanjayplp1@gmail.com (S.K.); 2 Department of Biotechnology, Guru Nanak Dev University, Amritsar 143005, India; 3 Academy of Scientific and Innovative Research, CSIR-Institute of Himalayan Bioresource Technology, Palampur, Himachal Pradesh 176061, India

**Keywords:** gene expression, interactome, microscopy, fertilization, self-incompatibility, transcriptome, tea

## Abstract

This study explicates molecular insights commencing Self-Incompatibility (SI) and CC (cross-compatibility/fertilization) in self (SP) and cross (CP) pollinated pistils of tea. The fluorescence microscopy analysis revealed ceased/deviated pollen tubes in SP, while successful fertilization occurred in CP at 48 HAP. Global transcriptome sequencing of SP and CP pistils generated 109.7 million reads with overall 77.9% mapping rate to draft tea genome. Furthermore, concatenated de novo assembly resulted into 48,163 transcripts. Functional annotations and enrichment analysis (KEGG & GO) resulted into 3793 differentially expressed genes (DEGs). Among these, de novo and reference-based expression analysis identified 195 DEGs involved in pollen-pistil interaction. Interestingly, the presence of 182 genes [PT germination & elongation (67), S-locus (11), fertilization (43), disease resistance protein (30) and abscission (31)] in a major hub of the protein-protein interactome network suggests a complex signaling cascade commencing SI/CC. Furthermore, tissue-specific qRT-PCR analysis affirmed the localized expression of 42 DE putative key candidates in stigma-style and ovary, and suggested that LSI initiated in style and was sustained up to ovary with the active involvement of *cs*RNS, SRKs & SKIPs during SP. Nonetheless, COBL10, RALF, FERONIA-rlk, LLG and MAPKs were possibly facilitating fertilization. The current study comprehensively unravels molecular insights of phase-specific pollen-pistil interaction during SI and fertilization, which can be utilized to enhance breeding efficiency and genetic improvement in tea.

## 1. Introduction

The purpose of pollination is fertilization and seed production to secure future survivability. Charles Darwin pioneered studies on the phenomenon of self-incompatibility in flowering plants “which are completely sterile with their own pollen, but fertile with that of any other individual of same species” [1]. This incapacity for self-pollination impeding self-fertilization is defined as self-incompatibility (SI). It is a genetically controlled mechanism that predominantly exists in flowering plants to overcome inbreeding depression and provides a high level of heterozygosity [2]. Self-incompatible plants have evolved genetic systems to prevent self-fertilization by recognition and rejection of pollen/pollen tube (PT) expressing the same allelic specificity either with pistils (pollen-pistil incompatibility) or ovular vicinity (ovular incompatibility/late-acting incompatibility), and post-fertilization mortality (post-zygotic incompatibility), inhibiting seed set [3]. Depending on the genetic control system, SI may be homomorphic or heteromorphic under the control of sporophytic or gametophytic conditions, and is categorized into three mechanisms, namely homomorphic sporophytic, homomorphic gametophytic and heteromorphic self-incompatibility [4,5]. 

Although SI has been widely studied in various angiosperms, nevertheless, molecular insights remained limited to Brassicaceae, Plantaginaceae, Rosaceae, Solanaceae and Papaveraceae. Among these, Brassicaceae possesses Sporophytic Self-Incompatibility (SSI), wherein, S-alleles of both the parents determine pollen’s compatibility [6]. The mechanism is controlled by a tightly linked allele of stigma-specific S-receptor kinase (SRK) and pollen-specific S-locus cysteine-rich protein (SCR)/s-locus protein 11 (SP11), often referred as S haplotype [7]. The pollen germination in plants with similar S-haplotype is obstructed by inhibition of a stigmatic compatibility factor, Exo70A1 by regulating the pollen hydration via water transport from papilla cells in stigma to facilitate the pollen germination [8].

The members of Plantaginaceae, Rosaceae & Solanaceae exhibit Gametophytic Self-Incompatibility (GSI), wherein the female determinant S-RNase acts as a cytotoxin inhibiting pollen with similar S-allele. A group of pollen determinant S-locus F-box (SLF/SCF complex) found in the vicinity of S-RNase gene in *Petunia* was controlling the pollen specificity commencing for either GSI or fertilization/cross-compatibility (CC) [9]. Furthermore, non-self S-RNase were targeted by pollen specific SCF complex and undergoes ubiquitin-mediated degradation inside the cross PT, while self S-RNase were not blocked by SCFs, subsequently degrading the pollen’s RNA and arresting PT growth [10]. Additionally, the roles of Pectin methyl esterase (PME) and pectin methyl esterase inhibitors (PMEI) were also reported in GSI in *Solanum species* [11]. Another type of GSI is reported in Papaveraceae, wherein Ca^2+^ mediated programmed cell death (PCD) occurs in self PT, preventing fertilization [12]. A recent transcriptome study in *Pyrus* species indicated a role of ATPase in SI through the calcium signaling pathway during the onset of pollination [13]. Moreover, late acting pre-zygotic SI or ovarian SI has been predominantly reported in Winteraceae, Theaceae, Malvaceae, Apocynaceae and Bignoniaceae families (eudicots); and Velloziaceae, Iridaceae, Amaryllidaceae and Xanthorrhoeaceae in monocots [4,14]. In some plant species like *Melaleuca alternifolia*, *Acacia retinodes* and *Theobroma cacao*, the PT normally grows up to ovary but failed to penetrate the ovule; while *Asclepiassyriaca* and *Spathodea campanulate* have been reported with post-zygotic LSI having abnormal/no seed set [15,16]. 

Tea (*Camellia sinensis* (L) Kuntze), indigenous to India and China, has been among the most profitable cash-crop across the globe. It is chiefly used as a ‘health/energy drink’ due to its ability to accumulate beneficial ingredients (mainly polyphenols) [17,18]. Belonging to family Theaceae, commercially important tea species have been classified into Chinese (*Camellia sinensis* var. *sinensis*), Assam (*Camellia sinensis* var. *assamica*) and Cambod (*Camellia sinensis* var. *assamica* subssp. *lasiocalyx*) forms of tea [19]. Due to tea’s high economic value, breeding efforts have been made for its genetic improvement, though these efforts are still incomplete due to certain bottlenecks such as a high outcrossing nature (allogamy), profuse phenotypic variation, perennial, long gestation periods, high inbreeding depression and self-incompatibility contributing to tremendous heterozygosity in tea [20,21]. Hence, conventional clonal propagation is preferred over natural propagation to maintain the quality lines. Considering the multiple advantages of cost-effective next-generation sequencing (NGS) technologies for molecular dissection of complex traits [22,23], an earlier study suggested involvement of the SCF complex and S-RNase during SI in the style [24]. Furthermore, investigations of ion components in self and cross pollinated pistils indicated the role of Ca^+2^ and K^+^ signal during SI [25]. Additionally, microscopy studies revealed LSI or ovarian sterility with pollen tube growth arrest in the SP ovary [26]. However, being a novel SI system, limited information is available regarding molecular insights regulating LSI response due to unidentified pollen/pistil factor having an important role in SI/CC reactions in tea [25,26]. In the current study, novel candidates involved in pollen-pistil interaction (LSI & fertilization) were identified by comparing the transcriptome of self-(SP) and cross-pollinated (CP) pistils in tea using high-throughput NGS technology. Furthermore, tissue-specific relative expression (style vs. ovary) of key genes provides a better understanding of the spatial transcriptional changes throughout the pistil during LSI. The results generated in this study elucidates important insights to understand the molecular mechanisms of LSI in light of fertilization in tea.

## 2. Results

### 2.1. Field Study and Microscopy Analysis 

**Figure 1:**
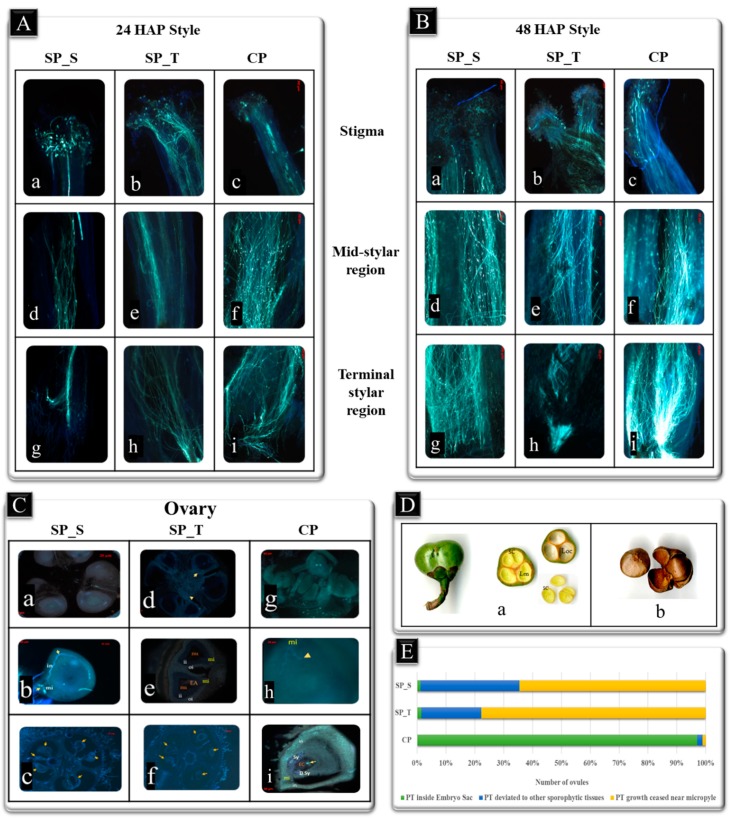
Pollen tube (PT) growth in self-pollinated pistil (SP) and cross-pollinated pistil (CP). (**A**) PT elongation in stigma (**a**–**c**), mid stylar region (**d**–**f**) and terminal stylar region (**g**–**i**) at 24 HAP SP and CP style. (**B**) PT growth in stigma (**a**–**c**), mid stylar region (**d**–**f**) and terminal stylar region (**g**–**i**) at 48 HAP SP and CP style. (**C**) PTs cessation (**a**,**d**,**e**) and deviation (**b**) at 48 HAP with abortive ovules (**c**,**f**) at 144 HAP in SP ovaries; PT (callose fluorescence) inside ovules (**g**), PTs infiltrating embryo sac (**h**), fertilized ovule with degenerated synergid (i) at 48 HAP CP ovaries. (**D**) 180 DAP fruit morphology and anatomy in CP pistil (**a**), 360 DAP seed morphology in CP pistil (**b**). nu represents nucellus, EA: Egg apparatus, in: integuments, ii: inner integument, oi: outer integument, mi: micropyle, sy: synergid, dsy: degenerated synergid, EC: Egg Cell, LEN: liquid endosperm, loc: locule, SC: seed coat (**E**) Graphical representation of microscopy inferences showing number fertilized ovules, number of ovules with PT deviation and number of ovules with PT cessation near micropyle at 48 HAP SP and CP pistils.

Pistil of both accessions (SA-6 and T78) possess wet type stigma with an ascending type style and syncarpous superior ovary [27]. The 24 h after Pollination (HAP) pistils were observed with PTs elongation up to the terminal region of style towards ovary in each case (Figure 1A). At 48 HAP Cross Pollinated pistils (CP), higher abundance of PT density and embryo sac with infiltrating PTs was observed in style and ovary, respectively (Figure 1B,C). In contrast, 48 HAP “Self-pollinated SA-6” (SP_S) and “Self-pollinated T78” (SP_T) exhibited less PT density in style with ceased/deviated PT towards integuments or other connective tissues in ovary (Figure 1B,C). A significant number of fertilized ovules (~97%) were recorded in reciprocal crosses of CP ovaries (SxT & TxS) at 48 HAP, while being insignificant in SP_S (1.1%) and SP_T (1.6%). However, a significant number of ovaries with abnormal PT behavior (ceased/deviated) near the micropyle in SP_S (98.8%) and SP_T (98.4%) was observed (Figure 1E and Table S1). Furthermore, a field study revealed ~60% fruit set at 180 Days after Pollination (DAP), and a seed set was observed at 360 DAP in both CP pistils (Figure 1D). In contrast, abortive ovules were also observed at 144 HAP in SP pistils [Figure 1C(c,f)]. Considering the microscopy inferences, 48 HAP was found to be an appropriate time to capture both fertilization and self-incompatible interactions for molecular analysis in our study. Additionally, a significant number of fertilization events with a strong positive correlation was recorded in both the reciprocal crosses (SxT and TxS) at 48 HAP, therefore, a single cross SxT of CP was utilized for transcriptome analysis.

### 2.2. Illumina Sequencing, Sequence Assembly and Functional Annotation

Based on microscopy inferences, cDNA libraries of self (SP) and cross-pollinated CP pistils were sequenced to surmise the global molecular insights of pollen tube-pistil interaction. Overall, 91.2 million filtered reads were obtained after quality filtering of 109.7 million raw reads (Figure S1). The de novo assembly of high-quality reads yielded 51,489 (average length: 543 bp; N_50_:719 bp) and 68,176 (average length: 776 bp; N_50_:960 bp) transcripts using CLC genomic workbench and TRINITY, respectively (Table S2). Furthermore, the assembled transcripts obtained from both assemblers were concatenated and clustered into 48,163 high-quality non-redundant (NR) transcripts. Additionally, reference-based assembly resulted in a 77.9% overall mapping rate of filtered reads (SP_S, 81.1%; CP, 77.9% and SP_T, 74.7%) with the tea draft genome [28]. 

To obtain the global functional insights of assembled transcripts, sequence homology search (BLASTx) was performed with various publicly available protein databases annotating 35,136 (73%), 33,017 (68.56%), 26,945 (55.9%) and 31,798 (66.02%) transcripts with NCBI’s nr, EggNOG, Swiss-Prot and TAIR10, respectively. The gene ontology (GO) annotation identified 23,996 transcripts assigned with 82,326 GO terms and classified them into the biological process (52%; 17 sub-categories), molecular function (22%; 7 sub-categories) & cellular component (26%; 8 sub-categories) (Table S3 and Figure S2a). Furthermore, a sequence search with Plant-TFDB resulted into 17,760 (36.56%) transcripts representing 58 transcription factors families. Among these, transcripts encoding basic helix-loop-helix transcription factor (bHLH) were the most abundant (2429 transcripts) followed by NAC (1663), MYB-related (1584), ERF (1278) and C2H2 (1038), (Figure S2b). Moreover, 378 pathways representing “metabolism” (44.5%), “genetic information & processing” (46.7%) and “signaling & cellular processes” (8.8%) exhibited significant enrichment in the KEGG pathway (Figure S2c).

### 2.3. Global Transcripts Expression Dynamics and Gene Ontology Enrichment Analysis 

To elucidate molecular insights and key regulators involved in SI and fertilization, differential gene expression (DGE) of self (SP_S and SP_T) and cross-pollinated (CP: SxT) pistils resulted into 3793 (SP_S vs. CP), 3530 (SP_T vs. CP) and 3423 (SP_S vs. SP_T) differentially expressed (DE) genes in de novo DGE analysis (*p*-value & FDR ≤ 0.05) (Table S4). While the reference genome based DGE yielded 1847 (SP_S vs. CP), 1919 (SP_T vs. CP) and 1298 (SP_S vs. SP_T) DE genes with *p*-value & FDR ≤ 0.05 (Table S5). Moreover, the gene ontology (GO) enrichment analysis revealed a maximum enrichment of GO categories in CP followed by SP_S and SP_T, respectively (Figure S3). The categories: “signal transduction”, “pollen-pistil interaction”, “embryonic and post-embryonic development” of biological process and “hydrolase”, “transferase”, “kinase”; “signal transducer & receptor activity”; and “proteasome & its regulatory complexes” of molecular function exhibited significantly higher enrichment in CP (Figure S4). However, “cell death” and “response to stress” showed significantly higher enrichment in SP pistils (SP_S and SP_T) (Figures S5c and S6c).

### 2.4. Phase Specific Differentially Expressed Transcripts Involved in Pollen-Pistil Interaction

**Figure 2:**
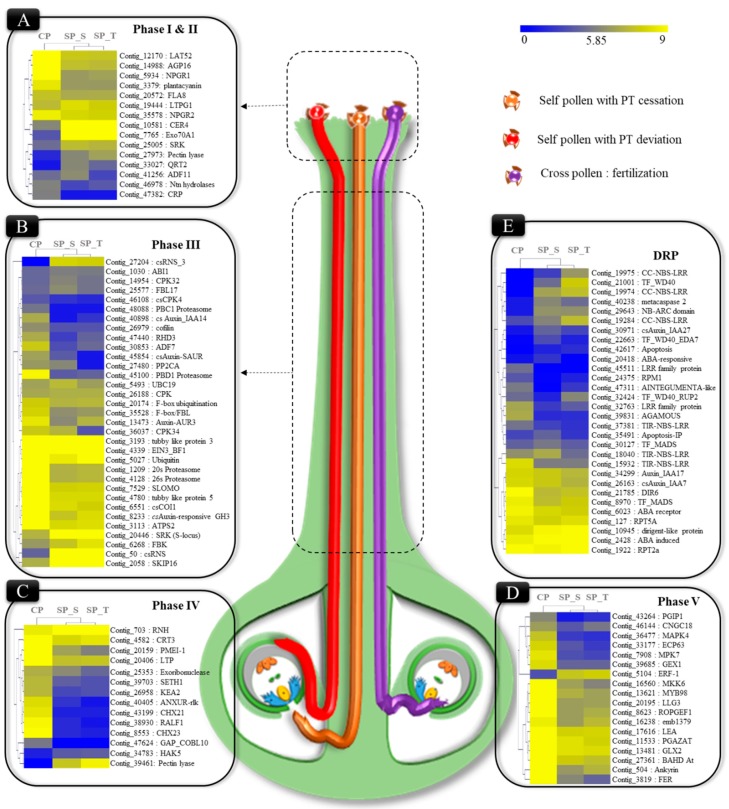
Schematic representation of PT elongation inside self and cross-pollinated pistil [Self PT: deviated (red) and ceased (brown), cross PT fertilization (purple)] as observed in microscopy, along with expression pattern of transcripts involved in different phases of pollen-pistil interaction. The heatmap represents expression pattern (log_2_ transformed FPKM) in yellow-blue scale. (**A**) Transcripts expression of genes involved in pollen germination and PT elongation (Phase I & II); (**B**) PT elongation in mid-stylar region and incompatible interactions (Phase III); (**C**) PT ovular guidance/rejection (Phase IV); (**D**) fertilization in cross-pollinated (Phase V) and; (**E**) disease resistance proteins.

Based on the global expression and GO enrichment analysis, 195 significantly DE transcripts (considering both de novo and reference-based DGE along with their functional relevance in SI & fertilization) were extracted and categorized into five phases during pollen-pistil interactions [29]. These phases include pollen germination in stigma region (Phase I), PT elongation in the upper stylar region (Phase II), PT elongation and incompatible interactions in the style transmitting tract (Phase III), PT ovular guidance and LSI interactions (Phase IV) and ovarian region encompassing genes involved in fertilization (Phase V) (Table S6). The transcripts corresponding to genes involved in the pollen germination of phase I (Exo70A1, SRK, CER4) along with gametophytic self-incompatibility of phase II-III [S-RNase (*cs*RNS), SKIP (ABI1 and EBF1), F-box like (FBL), Pectin lyase (polygalacturonase, PGLR; Exo-polygalacturonase, ExoPG)] and some disease resistance proteins (DRPs) were significantly upregulated in SP. Meanwhile, transcripts involved in normal PT elongation in style of Phase III (ANXUR-rlk, 26s proteasome, LAT52, Root hair defective (RHD), Lipid transfer proteins (LTP), Arabinogalactan protein (AGP)); PT-ovular guidance of phase IV [Rapid alkalization factor (RALF), COBL10, SETH, K^+^ transporters] and fertilization of phase V [FERONIA-rlk, LORELEI like glycoprotein (LLG), PMEI, GEX and ECP] along with auxin biosynthesis and auxin response factors (ARF) exhibited higher expression in CP (Figure 2).

### 2.5. Protein-Protein Interactome Network Analysis

**Figure 3:**
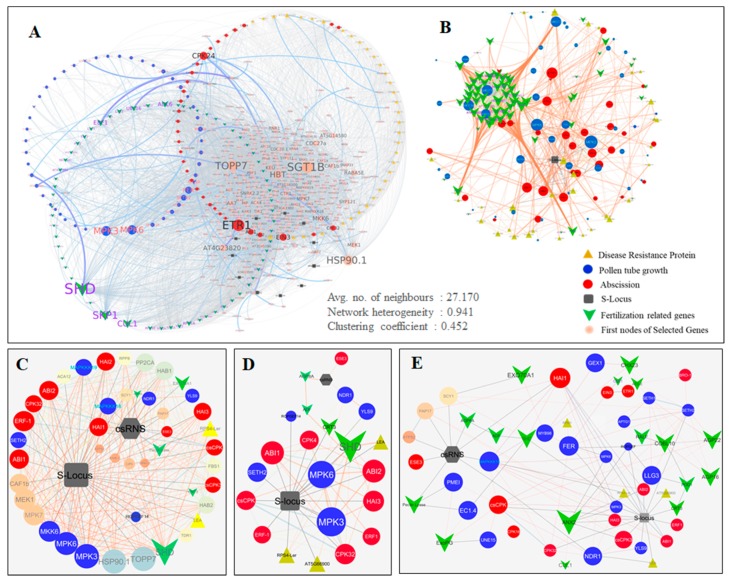
Predicted protein-protein interactome network of DE transcripts involved in fertilization or Self-Incompatibility in tea. (**A**) A major hub of 182 genes interacting with 343 first neighbors (6598 edges), (**B**) Co-expression network of 169 genes (1417 edges) extracted from 182 genes. (**C**) Gene specific predicted PPI-interactome network of S-Locus group (SRKs) and *cs*RNS *(C. sinensis).* (**D**) Direct interactions of S-locus related group and S-RNase. (**E**) Direct and indirect interactions of transcripts encoding genes involved in SI and Fertilization.

To identify the key regulatory genes and their involvement in complex signaling pathways during pollen-pistil interactions, a predetermined *At*PIN (*Arabidopsis thaliana* protein interaction network) was used [30]. The 195 DE transcripts showed direct interactions with 330 first neighbors (average number of neighbors: 27.170; network heterogeneity: 0.941 and clustering coefficient: 0.452). Interestingly, 182 nodes (1953 edges) were present in the major hub representing PT germination & elongation (67), S-locus related (13), Fertilization (43), disease resistance protein (DRPs, 30) and abscission (31) (Figure 3A and Table S7).

Furthermore, co-expression analysis revealed 148 genes (105 nodes in major hub) interacting with 211 first neighbors (2943 edges), displaying 129 incoming and 161 outgoing interactions (Figure 3B and Table S8). The degree of outgoing edges from node/gene (outgoing interactions) represents its regulatory function towards the node/gene receiving edges (incoming interaction) [31]. The intra-interactome network among five categories revealed that transcripts belonging to PT germination & elongation showed maximum outgoing interactions to the disease resistance proteins (DRP, 29) and abscission (16). Thus, transcripts involved in PT germination & elongation may have a role in pollen-pistil interaction by regulating DRPs and abscission-related genes. Furthermore, higher outgoing interactions of fertilization related genes with S-locus related (11), PT germination & elongation (57) and abscission (26) putatively suggested their major role in regulating PT growth to undergo fertilization or LSI. Higher outgoing interactions of S-locus related transcripts with the abscission-related genes, put forward their putative involvement in regulating PT abscission during LSI (Table 1). 

The direct interactions of S-locus related transcripts with the ovular guidance & fertilization, abscission, DRP, PT elongation; and indirect interactions with SI related transcripts (csRNS & Exo70A1) and ovular guidance cysteine rich proteins (RALF) also suggest their regulatory function during SI and CC. Furthermore, direct interaction of csRNS with AGP8A (autophagy 8A), peroxidase (PAP17), pectin lyase; and indirect interactions with actin depolymerization factor (ADF) & PMEI indicates its key role during incompatible interactions. Moreover, the ExoPG recorded direct interactions with the genes involved in PT growth arrest (PMEI & CPK24) may also have a role in self-incompatibility. A gene belonging to family receptor-like kinase (ANXUR-rlk) exhibited direct interactions with the genes involved in normal PT elongation and abscission, which probably suggests its role in normal PT elongation, and was also recorded with higher expression in CP. Moreover, the genes involved in ovular guidance GPI-Anchored proteins (COBL10) were found to be directly interacting with Rapid alkalization factor (RALF), arabinogalactan protein (AGP), Ca^++^ mediated signal transduction (csCPK), SETH and ROPGEF. This indicates their role in regulating PT ovular guidance for successful fertilization. Additionally, another receptor-like kinases (FERONIA-rlk) with significantly upregulated expression in CP, recorded direct/indirect interactions with fertilization related genes (ROPGEF, LLG, SETH, MPKs), thus it probably has a role in regulating fertilization (CC) (Figure 3C–E).

### 2.6. RNA-Seq Data Validation by qRT-PCR

**Figure 4:**
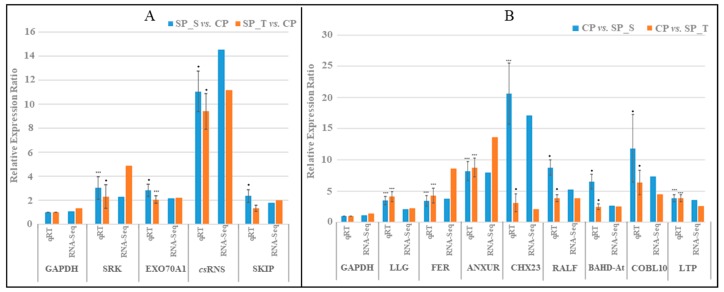
qRT-PCR validation of RNA-seq data using GAPDH as internal control. (**A**) Significantly upregulated SI related transcripts in SP pistils. (**B**) Significantly upregulated fertilization related transcripts in CP pistils. The bar represents standard deviation (SD) of relative expression for three replicated, and significance level is represented as stars: *p*-values (0.001, 0.01, 0.05) <=> symbols (“***”, “**”, “*”).

To confirm DGE inferences, qRT-expression validation of 12 key genes involved in pollen-pistil interaction during SP and CP showed a strong positive correlation with RNA-Seq expression data using GAPDH as an internal control (Figure 4A,B; Tables S9 and S10).

**Figure 5:**
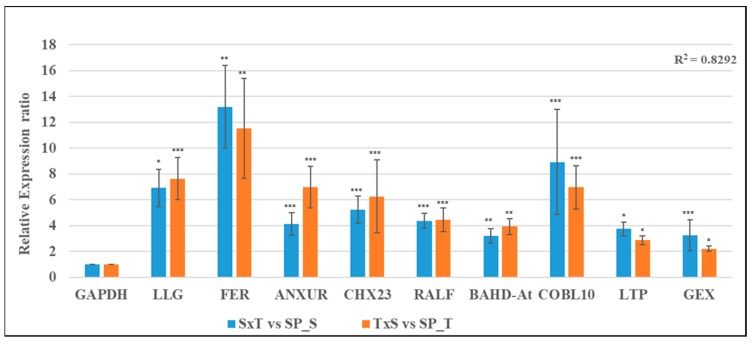
Relative expression analysis depicting strong positive correlation of reciprocal crosses (SxT & TxS) in CP pistils using GAPDH as internal control having strong positive correlation (*R*^2^ = 0.8292) between them. The bar represents SD of relative expression for three replicated experiments, significance level is represented as symbols (“***”, “**”, “*”) <=> *p*-values (0.001, 0.01, 0.05).

Interestingly, 9 of 12 fertilization related genes were significantly up-regulated in both CP with respect to their SP pistils (SxT vs. SP_S and TxS vs. SP_T), and recorded a strong positive correlation [*R* squared correlation coefficient (*R*^2^) = 0.8292] between the two reciprocal crosses of CP pistils (SxT & TxS) (Figure 5 and Table S10).

### 2.7. Tissue-Specific qRT-PCR Expression Dynamics during Pollen-Pistil Interaction 

**Figure 6:**
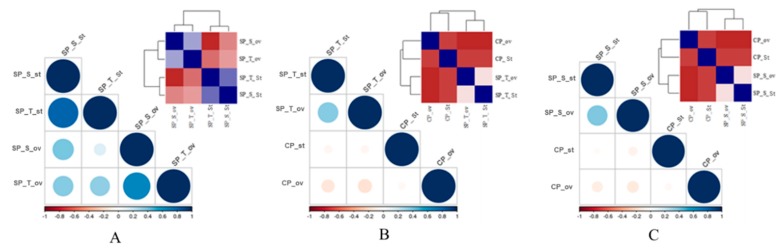
Tissue specific (style vs. ovary) and event specific (SP vs. CP) qRT-PCR expression correlation plot (correlation matrix and correlogram) of 42 key genes involved self-incompatibility and fertilization. (**A**) Tissue-specific correlation between self-pollinated tissues. (**B**) Event specific correlation between SP_S style and ovaries and (**C**) SP_T style and ovaries with respect to CP style and ovaries. The relative expression pattern is depicted in red-blue scale. Color intensity and size of the circle are proportional to the correlation coefficients. The legend color in the bottom represents the scale of correlation coefficients.

To study tissue and event specific expression, 42 key regulatory transcripts [pollen germination & elongation (9); ubiquitin-mediated protein degradation (6), ovular guidance (8), fertilization (12) and disease resistance (7)] were utilized for qRT-PCR relative expression analysis in style and ovary during SP and CP condition using GAPDH as an internal control (Table S9). A strong positive correlation in the expression pattern between SP genotypes in stylar (SP_S_style &. SP_T_style; *R*^2^ = 0.83) and ovary (SP_S_ovary & SP_T_ovary; *R*^2^ = 0.75) tissues possibly suggests a similar molecular behavior of incompatibility in both the SP pistils (Figure 6A and Figure S7A). However, an insignificant correlation in expression pattern between SP and CP possibly suggests a contrasting molecular mechanism commencing with SI and CC (Figure 6B,C; Figure S7B,C and Table S10).

**Figure 7:**
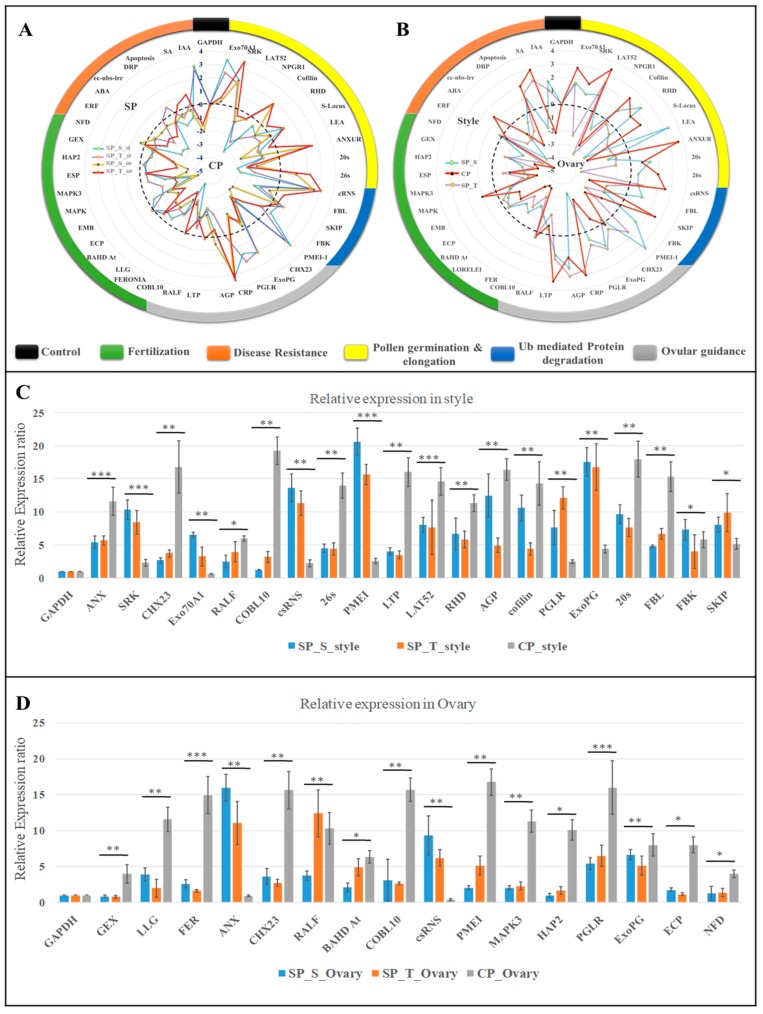
qRT-PCR expression analysis (log_2_ fold change) of 42 key genes using GAPDH as internal control in event specific (**A**) and tissue specific (**B**) manner. The positive values (periphery) represent genes upregulated in SP and style, while negative (center) represents upregulation in CP and ovaries. (**C**) Significant relative expression of genes in self and cross-pollinated style and; (**D**) ovaries with respect to unpollinated style and ovaries respectively. The error bar in the graph represents SD of relative expression for three replicated experiments and significance level is represented as symbols (“***”, “**”, “*”) <=> *p*-values (0.001, 0.01, 0.05).

The transcripts involved in SI (*cs*RNS & SRK) and pollen tube growth regulator (PMEI, PGLR, ExoPG) were upregulated in SP_style, while the transcripts participating in PT elongation (LAT52, cofilin, RHD, FBL) along with Ubiquitin mediated protein degradation (20s, 26s) [29] were highly expressed in CP_style. However, genes involved in PT-ovular guidance from stylar transmitting tract to ovule (RALF, LLG & COBL10) and fertilization (FER, GEX, hapless2, MAPKs and ECP) [29,32] exhibited upregulated expression in CP ovaries, and suggested a higher probability of PT ovular guidance commencing fertilization (Figure 7 and Figure S8). 

Interestingly, a significantly higher expression of *cs*RNS, PMEI, polygalactuonase (PGLR) and Exo-polygalaturonase (ExoPG) in SP style [33,34] suggests its role in lower PT growth rate in SP style. Furthermore, higher expression of ANXUR-rlk in CP_style and SP_ovary, possibly associated with normal PT elongation in CP_style, while deviation in SP_ovary. Additionally, up-regulated expression of PMEI in CP_ovary and SP_style might involve in slackening PT elongation to assist PT burst [35]. 

## 3. Discussion

The journey to fertilization is arbitrated by a series of complex signaling mechanisms from stigma to an ovary, wherein PT growth in style can stimulate changes within the ovary [36]. In the present study, the phenological, microscopic and genome-wide expressions forms of analysis have been comprehensively explored to unravel the complexity of SI/CC in tea. 48 HAP as implicated in this study was also appropriately reported with the pollen tubes elongation up to ovary commencing successful fertilization in earlier studies in tea [26]. The concatenated de novo assembly using two assemblers (CLC and TRINITY) resulted in high-quality non-redundant transcripts in this study [37]. Furthermore, ≥77 % mapping of reads with the reference genome of tea suggested quality transcriptome data in this study [28,38]. The higher enrichment of ‘signal transduction’, ‘post-embryonic development’ and ‘pollen-pistil interactions’ putatively suggests successful commencement of fertilization in CP (Figure S4), while, ‘cell death’ and ‘response to stress (endogenous and biotic)’ enrichment indicates the occurrence of SI in SP (Figures S5 and S6) [24]. Additionally, qRT validation of key genes of pollen-pistil interaction suggests the reliability of the RNA-Seq expression data. Significant abundance of fertilized ovules with a strong positive correlation in the expression pattern of fertilization related genes in reciprocal crosses (SxT & TxS) suggests the rare probability of unilateral incompatibility (UI) in tea [39]. 

Most of the SI related earlier studies have been focused on molecular dynamics between pollen and style, with limited attention given to the ovary specific events. Hence, the tissue-specific relative expression of 42 key candidates obtained in the current study were further investigated in a phase-specific manner (Phase I to V) [40] using stigma-style and ovary to gain a better understanding of the LSI response in the light of fertilization in tea. Considering an evolution of SI from pathogen defense mechanisms, the higher expression pattern of defense-related genes (CC-NBS-LRR; NB-ARC domain) and transcription factors (WD40) in SP suggests their possible involvement in incompatible PT arrest in tea [16,41].

### 3.1. Pollen Germination & PT Elongation (Phase I-III)

**Figure 8:**
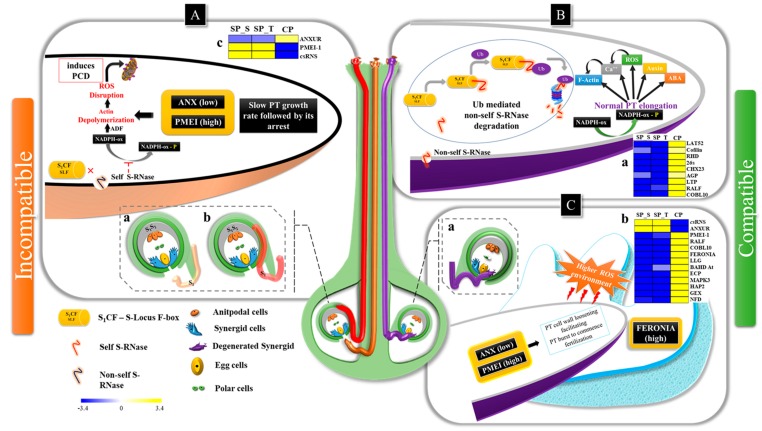
Summarized illustration representing self-incompatibility and cross-compatibility with tissue specific expression. The PT elongation within self and cross-pollinated pistil [Self PT: deviated (red) and ceased (brown), cross PT fertilization (purple)]. (**A**) Ceased (**a**) and deviated pollen tubes (**b**) representing incompatible interactions in style; (**c**) Heat map showing tissue specific qPCR expression of ANXUR-rlk, PMEI and csRNS in style revealing upregulated expression (yellow) of PMEI and csRNS in both SP coupled with downregulated expression (blue) of ANXUR-rlk; The self S-RNase (csRNS) in SP style inhibits phosphorylation of NADPH-ox, resultantly inducing programmed cell death (PCD) via depolymerization of actin cytoskeleton [44] (**B**) Normal PT elongation in style in CP as non-self S-RNase undergoes ubiquitin mediated protein degradation [10], (**a**) qPCR expression pattern showing up-regulated expression of genes involved in normal PT elongation in CP style. (**C**) Cross PT growth arrest followed by its burst within synergids commencing fertilization (**a**); qPCR expression pattern (**b**) of PT-ovular guidance and fertilization related genes exhibiting significantly up-regulated expression in CP ovaries. The lower expression of ANX coupled with higher expression of PMEI as observed in SP_style and CP_ovary suggests their putative role in inhibiting self-PT growth in SP style leading to SI and cross-PT inhibition in CP ovary to facilitate PT burst during fertilization. The yellow-blue scale represents fold change obtained in tissue specific relative expression analysis.

As reported in Brassicaceae, the pollens were physically adhered to stigmatic papilla cells by pollen coat proteins and hydrated via Exo70A1 in stigma, wherein pollen coat lipids assist in pollen hydration to undergo germination [42]. The higher expression of Exo70A1 in SP_style is possibly responsible for the wet type of stigma with higher stigma receptivity in SP than CP at 48 HAP [27]. Furthermore, lower PT density in SP_style can be attributed by an upregulated expression of SI related transcripts (csRNS, SRK, SKIP, ADF, pectin lyase, PGLR and Exo-PG) [43]. Moreover, *cs*RNS and S-locus related transcripts can be considered as key regulators due to their interactions with many compatibility and incompatibility factors in PPI network analysis. Additionally, indirect interaction of *cs*RNS with ADF suggests its possible role in programmed cell death (PCD) by depolymerization of actin cytoskeletons, hence arresting the self PT growth during GSI [10,44]. Considering an indicator of self-incompatibility, a significantly higher expression of Ca^+2^ transporters recorded in SP pistils may be responsible for higher concentration of Ca^+2^ ions in SP [25]. Nonetheless, the upregulated expression of transcripts involved in normal PT elongation (ANXUR-rlk, LAT52, cysteine rich proteins and RHD) and Ubiquitin-mediated S-RNase degradation (20s, 26s proteasome and SCF complex) may be attributed to higher PT density in the CP style (Figure 8A,B) [29,43].

### 3.2. PT-Ovular Guidance (PHASE IV & V)

The higher tissue-specific expression of ANXUR-rlk, cofilin and RHD involved in PT elongation may be correlated with PT deviation in SP ovaries [45,46]. Additionally, higher expression of *cs*RNS, PGLR, ExoPG and PMEI in SP (style & ovary) possibly associated with anomalous PT behavior, suggesting the initiation of LSI in style and its sustenance up to ovary [24]. Nevertheless, higher expression RALF, GPI-APs (COBL10 and LLG) and SETH in CP ovaries suggests their involvement during normal PT-ovular guidance (Phase IV) [42]. Furthermore, indirect interaction of S-locus (SRK) with COBL10 via SETH in PPI network probably suggests its regulation by SRK during compatible and incompatible interactions. Also, the interaction of SETH with GPI-APs (COBL10 & LLG), calcium channels (*cs*CPKs) and ROPGEF involved in downstream activation of NADPH-oxidase (increase ROS level) leads to PT-synergid cell burst, thereby commencing fertilization [25,32]. Additionally, COBL10 is reportedly involved in regulating PTs cell wall organization *via* pectin modifications by activating PMEI causing PT burst during fertilization and is governed by the ovular guiding signals [42]. The higher expression of PMEIs coupled with lower expression of ANXUR-rlk in SP_style and CP_ov suggests their putative role in inhibiting self-PT elongation in SP style leading to LSI, and cross-PT inhibition in CP ovary commencing successful fertilization [35,47] (Figure 8).

During fertilization (Phase V), the female “FERONIA dependent signaling pathway” is activated within synergid, while the male “ANXUR dependent signaling pathway” is deactivated in compatible PT [48]. In the current study, ANXUR-rlk and PME were found to be co-expressed in network analysis with significantly higher expression in CP_style and SP_ovary, which can be correlated with normal PT elongation by regulating PME. Meanwhile higher PMEI expression coupled with low ANXUR-rlk in CP ovary were possibly involved in the commencement of fertilization (Figure 8C) [35,47]. Additionally, the presence of FERONIA-rlk in the major hub having direct interactions with transcripts involved in the fertilization suggests its key regulatory role in commencing fertilization. Moreover, upregulated expression of FERONIA-rlk along with genes involved in double fertilization (GEX, HAP2 and BAHD acyltransferase) and transcription factor MAPK3 (Mitogen-activated Protein Kinase 3) can be correlated with higher frequency of fertilized ovules in CP ovaries as observed in microscopy [32]. 

## 4. Materials and Methods

### 4.1. Plant Material

Two self-incompatible tea accessions, SA-6 and Tukdah (T)-78 with high level of cross-compatibility [19,49] were selected in this study. These accessions were maintained at the CSIR-Institute of Himalayan Bioresource Technology, Palampur, India (1300 m altitude; 32°06′ N, 76°33′ E). Controlled pollination was carried out at the balloon stage (flowering) during October to December in three subsequent years (2013-2015). Enlarged and about to open floral buds with maximal stigmatic receptivity were emasculated, bagged and pollinated next day between 8:30 to 10:00 AM, followed by immediate re-bagging after pollination. The experimental analysis was performed in three combinations as “Self-pollinated SA-6 (SP_S)”; “Self-pollinated T78 (SP_T)” and “Cross-pollinated SA-6 x T78 and T78 x SA6 (CP)”. Pistils at 24 and 48 HAP were fixed for microscopy. A total 320 pollinated pistils (40 each for SP_S, SP_T and CP at 24 and 48 HAP) were collected for the microscopy, while some were leftover in the field to monitor the subsistent fruit and seeds set.

### 4.2. Microscopic Analysis

Twenty-four HAP and 48 HAP pistils were harvested and fixed in FAA fixative solution (Formaldehyde**:** Acetic acid:Alcohol::1:1:18) to target the PTs localization inside female gametophyte (pistil). Of the forty pistils, twenty each were used to trace the PTs inside stigma-stylar region using squash method and for targeting the PTs inside the ovary using microtome. For squash method, the pistils were fixed in F.A.A. for 24 h and stained using the aniline blue staining protocol [50]. Furthermore, 10 µm thin transverse sections of paraffin wax embedded ovaries were performed using microtome (Thermo Shandon Finesse microtome, Thermo Fisher Scientific, Waltham, MA, USA). Sections were mounted and stained using 0.1% aniline blue staining solution. The mounted stained samples and squashed samples were scanned and captured using Fluorescence microscope with AxioCam Zeiss MR Lenses (Oberkochen, Germany). Chi-square test was used to assess significance level of microscopy data to affirm the distinctness (*p* < 0.05) among collected samples.

### 4.3. RNA Extraction, cDNA Library Preparation And Illumina Sequencing

Based on microscopy inferences, 48 HAP pistils of SP_S, SP_T and CP (SxT) in ten biological replicates were collected and snap-frozen to liquid nitrogen for total RNA extraction using IRIS method [51]. The RNA was quantified on NanoDrop 2000 (Thermo Scientific, Waltham, MA, USA), and quality was assessed on 1% formaldehyde agarose gel (MOPS) and Agilent Bioanalyzer with RNA 7500 series II Chip (Agilent Technologies, CA, USA). The RNA samples with RIN (RNA Integrity Number) value greater than 8 and the final concentration of 4.0 µg were used for cDNA library preparation. 

Eight cDNA libraries in biological replicates SP_T (3), CP (3) and SP_S (2) were constructed using the illumina Truseq RNA Sample prep v2 LS Protocol (Illumina Inc., CA, USA). The libraries were quantified on Qubit 2.0 fluorometer (Invitrogen, USA), while quality was assessed using an Agilent 2100 Bioanalyzer (Agilent Technologies, CA, USA). The paired-end (PE) (2 × 72 bp) sequencing was performed using Illumina GAIIx. 

### 4.4. Quality Filtering, Sequence Assembly and Differential Expression Dynamics

The base calling and demultiplexing of raw data obtained from GAIIx run was performed using Illumina Casava 1.8.2 pipeline (http://support.illumina.com/). The demultiplexed raw reads were filtered using NGS QC Toolkit [52]. Filtered fastq reads were de novo assembled using both CLC Genomics Workbench 6.5 (CLC Bio-Qiagen, Aarhus, Denmark) and TRINITY RNA-Seq ver. 2.3.0 [53] with default parameters. Both of the assemblies were combined independently to optimize the coding region of transcriptome as discussed by Cerveau and Jackson (2016) [37]. The intra-assembly clustering of both the de novo assembled transcripts was performed using CD-HIT-ESTver4.6 clustering tool [54]. The unique transcripts derived from both the assemblies were concatenated and ORFs were detected using TransDecoder ver.3.0.1. These ORFs were further re-clustered based on their sequence similarity, yielding non-redundant high-quality transcripts. Individual sample reads were then separately mapped to the concatenated transcripts using Bowtie 2 and normalized to estimate transcript abundance and DE. The Transcript abundance was estimated using RPKM (Reads Per Kilobase of transcript per Million mapped reads) [55,56]. The differential gene expression between self-pollination (SP_S and SP_T) and cross-pollination (CP) events were estimated using the edgeR tool [57,58]. The p-values of DE transcripts were adjusted for multiple testing by the Benjamini-Hochberg false discovery rate (FDR) method [59]. The transcripts with FDR ≤0.05 and log_2_ FC ≥1 & ≤−1 were extracted for downstream analysis. Transcripts abundance (RPKM) was illustrated as a heatmap using MeV package v.4.9.0. Furthermore, with the advent of draft tea genome [28], reference-based DGE was also performed using Tuxedo reference genome based assembly pipeline with default parameters [60]. The sample-specific filtered reads were mapped to reference genome using TOPHAT ver2.1.0. Cufflink was used to assemble the transcriptome and estimate transcript abundance followed by Cuffmerge, to merge all the assemblies and estimate expression level. The DE transcripts between CP and SP conditions were compared using Cuffdiff. The TransDecoder ver.3.0.1 was used to extract the longest coding sequence using the merged GTF file obtained as an output from cuffmerge.

### 4.5. Transcripts Homology, Functional Classification and GO Enrichment Analysis

The de novo assembled transcripts were subjected to blastx analysis against the protein sequences in NCBI’s nr, Swiss-Prot, TAIR10, EggNOG v4.5 (http://eggnogdb.embl.de/), KEGG (http://www.kegg.jp/kegg/tool/annotate_sequence.html) and Plant Transcription Factor Database (http://planttfdb.cbi.pku.edu.cn/) considering *e*-value ≤ 1 × 10^−5^ to retrieve the top hits with functional attributes showing highest sequence similarity with the assembled transcripts. Gene enrichment was estimated using AgriGO toolkit. TAIR orthologous ID of DE transcripts was retrieved for GO enrichment using singular enrichment analysis (SEA) in AgriGO toolkit [61]. Plant GO slim was performed using Fischer statistical analysis (Hochberg-FDR adjustment cut-off <0.01) for optimal gene enrichment and represented in a hierarchical semantic similarity based scattered model and treemap (Figure S3). The in-silico enrichment analyses were computed using Bioconductor R package version 3.2.3. The GO terms were grouped into three categories: molecular function, biological processes, and cellular component. The over and under-represented GO terms were reduced and visualized on the Revigo tool using the Fisher-exact test. 

### 4.6. Protein-Protein Interactome Network Analysis

A protein-protein interactome network was built to identify key regulatory genes involved in incompatible and compatible interactions. The sequences homologues of DE transcripts were extracted from nr, TAIR and Swiss-Prot protein database and subjected to the STRING interactome public database for network analysis [62]. A correlation edge was considered as conserved when the selected tea genes had a significant correlation edge with its respective orthologs in the *Arabidopsis thaliana* PPI network. First neighbors of the mapped IDs were selected for predicting their interaction. Subsequently, a regulatory network was built based on phylogenetic co-occurrence, the number of directed edges, homology and co-expression of values. This network was visualized on Cytoscape ver. 3.4.0 [63]. Genes of selected categories were represented in circular layouts using a number of directed edges as an attribute. 

### 4.7. RNA-Seq Expression Pattern Validation Using Real-Time PCR

Differential Gene expression of 12 DE transcripts from RNA-Seq data were validated utilizing Real time PCR (RT-PCR). The RNA of whole pistil from each SP_S, SP_T and CP was considered in RNA-Seq validation as utilized in RNA-Seq analysis. Additionally, RNA from SxT & TxS pistils was also extracted to scrutinize the expression pattern of 9 fertilization related genes between two reciprocal crosses (Table S9). The first strand cDNA was synthesized using 2 µg of total RNA by Revert Aid First strand cDNA synthesis kit (Thermo Scientific, USA). Gene-specific primers from selected transcripts were designed with BatchPrimer3 (http://probes.pw.usda.gov/batchprimer3/). Reactions were performed in 20 µL reaction volume containing 200 ng template cDNA with FG-POWER SYBR^®^ Green PCR Master Mix Applied Biosystem (Foster City, CA, USA) and gene-specific primers (Table S9) in StepOne™ Real-Time PCR System (Applied Biosystem). Specific GAPDH primers were used as an internal control. The expression analysis of all the genes were performed in three replicates and relative expression was calculated using comparative Ct values [59,64]. 

### 4.8. Tissue-Specific Transcript Expression Analysis Using qRT-PCR 

42 putative key candidate genes involved in compatible/incompatible interactions were selected based on their functional annotation, enrichment and PPI network analysis to assess tissue specific (style vs. ovary) and event specific (SP vs. CP) relative expression analysis using qRT-PCR. Total RNA was extracted from both 48 HAP style and ovaries, separately from each SP_S, SP_T and CP along with their respective controls (un-pollinated style and ovary) using IRIS method [51]. The cDNA preparation and qRT-analysis were performed as mentioned in the previous section (Section 4.7) using GAPDH as a reference gene (Table S9). The expression analysis of all the genes were performed in three replicates and relative expression was calculated using comparative Ct values [59,64]. The relative expression ratio of SP and CP, style and ovaries were obtained with respect to unpollinated style and ovaries. Furthermore, ovaries and CP were considered as control in tissue specific and event specific fold change analysis respectively. Pearson’s correlation coefficient along with their significance were computed based on candidate genes specific relative expression ratio to find tissue specific and event specific correlation and were plotted using the R package. 

## 5. Conclusions

The current study provides a comprehensive atlas of genes and pathways involved in pollen pistil interaction leading to LSI in light of fertilization in tea. Combined inferences drawn based on microscopy, genome-wide transcriptome, interactome network and tissue specific qRT-expression analysis suggests a pre-zygotic type of LSI, which probably initiates in style and sustains up to ovary with the active involvement of potential candidates belonging to categories cysteine-rich proteins (RALF), receptor-like kinases (FER-rlk, ANXUR-rlk), GPI-Aps (COBL10, LLG), enzyme (csRNS, PME & PMEI) and transcription factors (MAPK). The valuable genomic resources and putative master regulators obtained in this study will promote a better understanding of the molecular mechanism of pollen-pistil interaction that commences LSI and fertilization in tea. These resources can be employed to enhance breeding efficiency and genetic improvement in tea and other perennial plant species. 

## Figures and Tables

**Table 1:** Intra-interactome network analysis among five categories showing a number of outgoing and incoming interactions.

Outgoing Interactions	Incoming Interactions				
	PT Germination & Elongation	S-Locus Related	Fertilization	DRP	Abscission
PT germination & elongation	**67**	3	26	29	16
S-locus related	2	**11**	5	3	8
Fertilization	57	11	**43**	5	26
DRP	7	2	6	**30**	4
abscission	27	10	20	2	**31**

## Data Availability

The raw reads were deposited to NCBI SRA database with the following accession numbers: SRR7037029, SRR7037030, SRR7037031, SRR7037032, SRR7037033, SRR7037034, SRR7037035, SRR7037036.

## Supplementary Materials

The following are available online at http://www.mdpi.com/1422-0067/20/3/539/s1, Figures S1–S8; Tables S1–S10. Figure S1: Quality check and filtering of RNA-seq Data. [a] Overall Filtering of Data. [b] Sample wise Filtering of Data. Figure S2: (a) Overall GO annotation of transcripts categorized into cellular component, biological process and molecular function, (b) Transcripts annotation with 58 Plant transcription factors represented in the form of scattered plot, top 20 TF represented in the form of pie chart and (c) KEGG pathway classification of overall transcripts. Figure S3: Scattered plot of GO terms associated with upregulated transcripts in (a) SP_S, (b) SP_T and (c) CP condition exhibiting clusters representatives. The matrix was created using pairwise semantic similarities of GO terms. The semantically similar GO terms were clustered together. Revigo tree map representation of Upregulated transcripts in (d) SP_S, (e) SP_T and (f) CP condition showing GO enrichment. The treemap was built using absolute log10 P-value, the blocks with larger area showing categories with higher GO enrichment and the blocks with similar semantic values are represented with similar colours. Figure S4: GO enrichment analysis of upregulated transcripts in CP condition [a] Biological Processes; [b] Molecular Function; [c] Cellular Components. Figure S5: GO enrichment analysis of upregulated transcripts in SP_S condition [a] Biological Processes; [b] Molecular Function; [c] Cellular Components. Figure S6: GO enrichment analysis of upregulated transcripts in SP_T condition [a] Biological Processes; [b] Molecular Function; [c] Cellular Components. Figure S7: Correlation plot representing correlation between event specific and condition specific RT expression analysis (a) Correlation among SP_S_ss, SP_T_ss, SP_S_ov and SP_T_ov; (b) Correlation among SP_S_ss, CP_ss, SP_S_ov and CP_ov; (c) Correlation among SP_T_ss, CP_ss, SP_T_ov and CP_ov. The distribution of each variable is shown on the diagonal with bottom showing bivariate scatter plots with a fitted line top, showing the value of the correlation. The significance level is represented as stars, each significance level is associated to a symbol: p-values(0, 0.001, 0.01, 0.05, 0.1, 1) <=> symbols(“***”, “**”, “*”, “.”, " “). Figure S8: Graph representing log2 fold change expression of 42 key genes using GAPDH as internal control in event specific (A) and tissue specific (B) manner. The error bar represents SD between three replicated samples. Table S1: Chi-square estimation of microscopy results. Table S2: Summary details of assembled sequences produced after filtered reads de novo assembly using both CLC work bench and TRINITY. Table S3: Annotation of assembled sequences (Transcripts) based on five public databases TAIR, swissprot, nr, KOG (EGGNOG) and Transcription Factors. Table S4: Differential Gene Expression extracted from de novo assembly using EdgeR tools. Tab 1 showing DGE between SP_S and CP, Tab 2 DGE between SP_T and CP. Table S5: Differential Gene Expression extracted from tea reference genome using tuxedo pipeline. The fold change was calculated between SP_S vs CP and SP_T vs CP using cuffdiff. Table S6: 408 Differentially expressed transcripts encapsulating 195 genes related to fertilization and incompatibility categorized into five different phases. Table S7: Gene specific interactome network analysis of differentially expressed genes. Table S8: Gene specific coexpression analysis of differentially expressed genes. Table S9: Primer details of selected transcripts sequences to assess relative expression analysis using quantitative Real Time-polymerase chain reaction (qRT-PCR) analysis. Table S10: RNA-Seq validation of DGE with raw Ct values of reciprocal crosses and tissue specific relative expression analysis with significance test.

## Abbreviations

SI	Self-incompatibility
LSI	Late-acting gametophytic self-incompatibility
CC	Cross-compatibility (Fertilization)
SP	Self-Pollinated
CP	Cross-pollinated
PT	Pollen tube
HAP	Hours after pollination
DAP	Days after pollination
KEGG	Kyoto encyclopedia of genes and genomes
GO	Gene ontology
DGE	Differential gene expression
NGS	next generation sequencing
SLF/SCF	S-locus F-box protein
SRK	S-receptor kinase
CPK	Calcium-dependent protein kinases
TLP	Tubby like proteins
RHD	Root hair defective
PMEI	Pectin methylesterase inhibitor
GEX	Gamete expressed
ARF	auxin response factors
DRP	Disease resistance proteins
RALF	Rapid alkalization factors
LLG	LORELLEI like glycoprotein
GPI-Ap	Glycosylphosphatidylinositol anchored protein
CRP	Cystein rich protein
qRT-PCR	Quantitative Real-Time PCR
