# Supplementary material for: Global Transcriptional Insights of Pollen-Pistil Interactions Commencing Self-Incompatibility and Fertilization in Tea [Camellia sinensis (L.) O. Kuntze]
Source: Int J Mol Sci. 2019 Jan 28;20(3):539. doi: 10.3390/ijms20030539 (PMC6387076; doi:10.3390/ijms20030539)
Supplement: Supplementary file 1 [file ijms-20-00539-s001.zip › Supplementary Information.doc]

**Supplementary Material**

**Article**

**Global transcriptional insights of pollen-pistil interactions revealed key candidates commencing self-incompatibility and fertilization in tea [Camellia sinensis (L.) O. Kuntze]**

**Romit Seth**^1,2^**, Abhishek Bhandawat**^1^**, Rajni Parmar**^1,3^**, Pradeep Singh**^1,2^**, Sanjay Kumar**^1,3^**, Ram Kumar Sharma**^1,3^*****

^1^Biotechnology Department, CSIR-Institute of Himalayan Bioresource Technology, Palampur, Himachal Pradesh, India, 176061

^2^Department of Biotechnology, Guru Nanak Dev University, Amritsar, 143005, India

^3^Academy of Scientific and Innovative Research, New Delhi, India, 176061

***Correspondence:**

Dr Ram Kumar Sharma
Biotechnology Division
CSIR-Institute of Himalayan Bioresource Technology
Palampur (H.P)
India
Email: rksharma.ihbt@gmail.com, ramsharma@ihbt.res.in

**Figure S1:** Quality check and filtering of RNA-seq Data. [a] Overall Filtering of Data. [b] Sample wise Filtering of Data.

**Figure S2:** (a) Overall GO annotation of transcripts categorized into cellular component, biological process and molecular function, (b) Transcripts annotation with 58 Plant transcription factors represented in the form of scattered plot, top 20 TF represented in the form of pie chart and (c) KEGG pathway classification of overall transcripts.

**Figure S3:** Scattered plot of GO terms associated with upregulated transcripts in (a) SP_S, (b) SP_T and (c) CP condition exhibiting clusters representatives. The matrix was created using pairwise semantic similarities of GO terms. The semantically similar GO terms were clustered together. Revigo tree map representation of Upregulated transcripts in (d) SP_S, (e) SP_T and (f) CP condition showing GO enrichment. The treemap was built using absolute log_10_ P-value, the blocks with larger area showing categories with higher GO enrichment and the blocks with similar semantic values are represented with similar colours.

**Figure S4:** GO enrichment analysis of upregulated transcripts in CP condition [a] Biological Processes; [b] Molecular Function; [c] Cellular Components.

**Figure S5:** GO enrichment analysis of upregulated transcripts in SP_S condition [a] Biological Processes; [b] Molecular Function; [c] Cellular Components.

Figure S6: GO enrichment analysis of upregulated transcripts in SP_T condition [a] Biological Processes; [b] Molecular Function;

[c] Cellular Components.

**Figure S7**: Correlation plot representing correlation between event specific and condition specific RT expression analysis (a) Correlation among SP_S_ss, SP_T_ss, SP_S_ov and SP_T_ov; (b) Correlation among SP_S_ss, CP_ss, SP_S_ov and CP_ov; (c) Correlation among SP_T_ss, CP_ss, SP_T_ov and CP_ov. The distribution of each variable is shown on the diagonal with bottom showing bivariate scatter plots with a fitted line top, showing the value of the correlation. The significance level is represented as stars, each significance level is associated to a symbol: p-values(0, 0.001, 0.01, 0.05, 0.1, 1) <=> symbols(“***”, “**”, “*”, “.”, " “)

**A.**

**Figure S8**: Graph representing log2 fold change expression of 42 key genes using GAPDH as internal control in event specific (A) and tissue specific (B) manner. The error bar represents SD between three replicated samples.

**B.**

**Table S1: Chi-square estimation of microscopy results.** Chi-square = 936.8602, degree of freedom = 6; two tailed p-value < 0.0001; Yates' correction for continuity = 925.641 (p-value < 0.0001). Obs. = no. of ovules; Exp. = expected number of ovules; HAP = Hours After Pollination.

|  |  |  |  | **Samples** |  |  |  |
| --- | --- | --- | --- | --- | --- | --- | --- |
| **Pollen Tube (PT) location inside ovary at 48 HAP** | |  | **SP_S** | **CP (SxT)** | **CP (TxS)** | **SP_T** |  |
| **CC** | **PT infiltrating Embryo Sac** | **Obs.** | **3** (1.1%) | **267** (97.1%) | **236**  (95.1%) | **4** (1.6%) | **510** |
|  |  | **Exp.** | *131.3* | *134.73* | *121.5* | *122.48* |  |
| **SI** | **PT deviation within ovary** | **Obs.** | **92** (34.3) | **5**  (1.8%) | **4** (1.6%) | **84** (33.6%) | **185** |
|  |  | **Exp.** | *47.63* | *48.87* | *44.07* | *44.43* |  |
|  | **PT ceased near micropyle** | **Obs.** | **173** (64.5%) | **3**  (1.1%) | **8** (3.2%) | **162** (64.8%) | **346** |
|  |  | **Exp.** | *89.08* | *91.4* | *82.43* | *83.09* |  |
|  |  |  | **268** | **275** | **248** | **250** | **1041** |

**Table S2** Assembly statistics of transcriptomic reads obtained from illumina sequencing.

| Assembly Statistics | | |
| --- | --- | --- |
| Raw Reads | 10,90,00,014 | |
| Filtered Reads | 9,11,80,308 | |
|  | **CLC** | **TRINITY** |
| No. of assembled transcripts/contigs | 51,489 | 68,176 |
| Minimum sequence length | 182 | 301 |
| Maximum sequence length | 8454 | 7655 |
| N_50_ length | 719 | 960 |
| Average sequence length | 543 | 776 |
|  |  |  |

**Table S9**: Details of primer sequences of selected contigs used for quantitative Real Time-polymerase chain reaction (qRT-PCR) analysis.

| **S.no.** | **Contig. ID** | **Gene** | **Primer Sequence** | **Ta °C** | **Remark** |
| --- | --- | --- | --- | --- | --- |
| **1** | Contig_573 | GAPDH_HK | Fwd. 5'-CTCCATCACAGCCACACAAA-3' | 60 | TS, RV |
|  |  |  | Rev. 5'-GCACCTTACCAACAGCCTTG-3' |  |  |
| **2** | Contig_18234 | CC-NBS-LRR | Fwd. 5'-CATTCGGCGTTTGGTCATTAG-3' | 60 | TS |
|  |  |  | Rev. 5'-TCTCAATCTTTCTGCGGGCTTA-3' |  |  |
| **3** | Contig_8837 | DRP | Fwd. 5'-GTCCATTTCACTCATTTCACCTG-3' | 60 | TS |
|  |  |  | Rev. 5'-GCGAAGGCGGTTGCTTAT-3' |  |  |
| **4** | Contig_12170 | LAT52 | Fwd. 5'-GCCCAATCCCACGAAGAG-3' | 62 | TS |
|  |  |  | Rev. 5'-TCAGTTCCACCTCAGCACCTT-3' |  |  |
| **5** | Contig_25005 | SRK *_(N. sylvestris)_* | Fwd. 5'-TCAGAGCGAAACCCATACG-3' | 60 | TS, RV |
|  |  |  | Rev. 5'-AGAAGCAACACACCGAAGC-3' |  |  |
| **6** | Contig_5934 | NPGR1 | Fwd. 5'-AGCCCTTGTTGCCTTCACA-3' | 62 | TS |
|  |  |  | Rev. 5'-GTGGGTTCTAATCGCAGAGCA-3' |  |  |
| **7** | Contig_7765 | Exo70A1 | Fwd. 5'-CGGTCTTGAGTGCGACGAG-3' | 61 | TS, RV |
|  |  |  | Rev. 5'-ATTGCGTTGGGTGGAGAAAG-3' |  |  |
| **8** | Contig_1019 | PGLR | Fwd. 5'-AGTCGGGAGAGGCGGGTAT-3' | 58 | TS |
|  |  |  | Rev. 5'-GTCAGCGTGGGAGCCATAAT-3' |  |  |
| **9** | Contig_12854 | Exo-PG | Fwd. 5'-CCACATTTTCACAAGGGTTTCC-3' | 59 | TS |
|  |  |  | Rev. 5'-GCACATCAAAGGAGCCATCAC-3' |  |  |
| **10** | Contig_3964 | cofilin | Fwd. 5'-GCTGAAGAGGAAGAAGGTTTATCG-3' | 60 | TS |
|  |  |  | Rev. 5'-AGGCAAGGACGCAGTGAAAT-3' |  |  |
| **11** | Contig_39083 | Apoptosis | Fwd. 5'-TCGCCGTATTTCAGGAGTGG-3' | 62 | TS |
|  |  |  | Rev. 5'-CAAAACCAGAGCCGAGACCTT-3' |  |  |
| **12** | Contig_20406 | LTP | Fwd. 5'-AGCAGCCAAAGTTCCACCAAC-3' | 64 | TS, RV, RC |
|  |  |  | Rev. 5'-CCAAAGGCGACAGCAGGAC-3' |  |  |
| **13** | Contig_1214 | CRP | Fwd. 5'-TGTCATAGGTCCAACACCCACTT-3' | 61 | TS |
|  |  |  | Rev. 5'-ATGCCCAAAACTCACCACAAG-3' |  |  |
| **14** | Contig_14988 | AGP | Fwd. 5'-GAATGGGATGGGGAAAGGAAT-3' | 58 | TS |
|  |  |  | Rev. 5'-GAAAAGGCGAACAATGGCACT-3' |  |  |
| **15** | Contig_6268 | FBK | Fwd. 5'-TGGGCGTCTAATGAACCTGA-3' | 60 | TS |
|  |  |  | Rev. 5'-CAATCTCTTGCCACTCCTTCC-3' |  |  |
| **16** | Contig_35528 | FBL | Fwd. 5'-TCTGAGGTCGGCACACGAT-3' | 62 | TS |
|  |  |  | Rev. 5'-GGGCAACCACTTAGCAACTCC-3' |  |  |
| **17** | Contig_1209 | 20S proteasome | Fwd. 5'-GTGGGACTGTTGTTGGCATAAA-3' | 61 | TS |
|  |  |  | Rev. 5'-CGATGAACGGAGTGGATTCTG-3' |  |  |
| **18** | Contig_2058 | SKIP | Fwd. 5'-TTGAAAGTGGAGATGGGATTG-3' | 58 | TS, RV |
|  |  |  | Rev. 5'-AGCGTGAGCAAGAGGTTCC-3' |  |  |
| **19** | Contig_948 | MAPK | Fwd. 5'-GCTTCTTTCCCGCCTTCTCTG-3' | 62 | TS |
|  |  |  | Rev. 5'-CGCTGCCATTCGGTTTGAG-3' |  |  |
| **20** | Contig_10640 | SA binding | Fwd. 5'-GAAAGCAGTGAGGAAGACAGCA-3' | 61 | TS |
|  |  |  | Rev. 5'-GAGCCGTTGATGGAGGTCAT-3' |  |  |
| **21** | Contig_20446 | SRK *_(S. squalidus)_* | Fwd. 5'-ACGAATCACAAGCAAACACCA-3' | 59 | TS, RV |
|  |  |  | Rev. 5'-GCCAAACAAAATCTCAAGCATC-3' |  |  |
| **22** | Contig_18574 | MAPK3 | Fwd. 5'-TGGAAAAACCATTGCTAACTGC-3' | 60 | TS |
|  |  |  | Rev. 5'-GGCACACCCACTGAATCG-3' |  |  |
| **23** | Contig_4128 | 26S Proteasome | Fwd. 5'-TGGCTGCGAGACTTTACTTCT-3' | 60 | TS |
|  |  |  | Rev. 5'-GTTTCCTGACCCAACTCATCA-3' |  |  |
| **24** | Contig_10250 | RHD  (Root hair defective) | Fwd. 5'-GCTGAGGAGGGAACGAAGACT-3' | 62 | TS |
|  |  |  | Rev. 5'-CAGACGAAGCAGTGGATGACA-3' |  |  |
| **25** | Contig_2428 | ABA | Fwd. 5'-GCTCTGGTTGTTCAGGAGGTGT-3' | 61 | TS |
|  |  |  | Rev. 5'-TGCGGTTTCGTGTTCTATTCG-3' |  |  |
| **26** | Contig_15186 | PMEI1 | Fwd. 5'-TTCTCTCCTCACCCTCCTCTC-3' | 61 | TS |
|  |  |  | Rev. 5'-GCTTGTTCGGATGTAGTTGGT-3' |  |  |
| **27** | Contig_40405 | ANXUR | Fwd. 5'-TGGGTGATGTGTTGTGGAATC-3' | 59 | TS, RV, RC |
|  |  |  | Rev. 5'-CGCTTGTTGAAGTTTGCTTTG-3' |  |  |
| **28** | Contig_15107 | NFD | Fwd. 5'-CTGGCGGTGATGAACAATCTG-3' | 62 | TS |
|  |  |  | Rev. 5'-CCGAACCCGAAACGATGC-3' |  |  |
| **29** | Contig_8233 | IAA | Fwd. 5'-GCCAAACAACACTGAGCCAAC-3' | 59 | TS |
|  |  |  | Rev. 5'-CACGAGTTACGCCGATACGAA-3' |  |  |
| **30** | Contig_8553 | CHX23 | Fwd. 5'-TGCCCATCCAGCCTCTGAT-3' | 61 | TS, RV, RC |
|  |  |  | Rev. 5'-CCAATGACAGCAATCTCACCAA-3' |  |  |
| **31** | Contig_1948 | BAHD acyltransferase | Fwd. 5'-ATGAATGCCAGGGAAGAGC-3' | 57 | TS, RV, RC |
|  |  |  | Rev. 5'-TGGTGGAGATTAGGGTTTGCT-3' |  |  |
| **32** | Contig_19199 | RALF | Fwd. 5'-CAGCCGTGTCATCCTTCTCAT-3' | 62 | TS, RV, RC |
|  |  |  | Rev. 5'-CAGCCTCGGAAACCATCATC-3' |  |  |
| **33** | Contig_19636 | ERF | Fwd. 5'-CAACGGACAAACAGTAGAAACACA-3' | 61 | TS |
|  |  |  | Rev. 5'-GAGGTGACGCTGAGGAGAGAG-3' |  |  |
| **34** | Contig_20195 | LORELEI | Fwd. 5'-ACATCAACCTCTACGGGAAATACC-3' | 60 | TS, RV, RC |
|  |  |  | Rev. 5'-GGAACACCACTTTTGGCGATA-3' |  |  |
| **35** | Contig_48344 | ESP | Fwd. 5'-CGAACGATACATTTTTAGGGCAAC-3' | 61 | TS |
|  |  |  | Rev. 5'-GGCTCGGACTTGTGTGATAGG-3' |  |  |
| **36** | Contig_16238 | EMB | Fwd. 5'-ATGGAGGGTGGAGGAGGTG-3' | 63 | TS |
|  |  |  | Rev. 5'-CCAGAAACGGGGTGAGAAAGT-3' |  |  |
| **37** | Contig_33177 | ECP | Fwd. 5'-ACCACCACCACCACCACCAC-3' | 65 | TS |
|  |  |  | Rev. 5'-AACAGGGGGACGGCAGGAT-3' |  |  |
| **38** | Contig_1649 | LEA | Fwd. 5'-TACAGAAACGGCGACCAAA-3' | 58 | TS |
|  |  |  | Rev. 5'-ATCACTCTCCCCACTCTCCTC-3' |  |  |
| **39** | Contig_14636 | FERONIA-rlk | Fwd. 5'-CGACAACTCCGTCACCAGAAT-3' | 60 | TS, RV, RC |
|  |  |  | Rev. 5'-TCGTTACCACATCATCCATCG-3' |  |  |
| **40** | Contig_25245 | GEX | Fwd. 5'-GACCGCTTCTCTTCACCTGCT-3' | 65 | TS, RC |
|  |  |  | Rev. 5'-ACACCACACCACCAACTCCAC-3' |  |  |
| **41** | Contig_50 | *cs*RNS | Fwd. 5'-TTGTGCTGAGTCTGTCTTTGACC-3' | 58 | TS, RV |
|  |  |  | Rev. 5'-GCAGTGGTGAGGGCTTGAA-3' |  |  |
| **42** | Contig_47624 | COBL10 | Fwd. 5'-TTCAACGGCACCAAACTGC-3' | 58 | TS, RV, RC |
|  |  |  | Rev. 5'-AAACGAAATCACCGACTGCTG-3' |  |  |
| **43** | Contig_40481 | HAP2 | Fwd. 5'- CTGTCTCCGATTTCCAGGTGA-3' | 57 | TS |
|  |  |  | Rev. 5'- GCTTGCTGTTGGCTTATTTGC-3' |  |  |

***T_a_°C****: annealing temperature; TS: Tissue specific expression analysis; RV: RNA-Seq expression validation; RC: Reciprocal crossing*

**qRT-PCR conditions:**

The PCR amplification program was as follows:

95°C for 10min; 40 cycles of 95°C for 30s, annealing temperature (T_a_°C) for 30s and 72°C for 30s; followed by a melting-curve program of 95°C for 1min, 60°C for 30s and 95°C for 30s

**SUPPLEMENTARY LEGENDS**

**Supplementary Figures**

**Figure S1:** Quality check and filtering of RNA-seq Data. [a] Overall Filtering of Data. [b] Sample wise Filtering of Data.

**Figure S2:** (a) Overall GO annotation of transcripts categorized into cellular component, biological process and molecular function, (b) Transcripts annotation with 58 Plant transcription factors represented in the form of scattered plot, top 20 TF represented in the form of pie chart and (c) KEGG pathway classification of overall transcripts.

**Figure S3:** Scattered plot of GO terms associated with upregulated transcripts in (a) SP_S, (b) SP_T and (c) CP condition exhibiting clusters representatives. The matrix was created using pairwise semantic similarities of GO terms. The semantically similar GO terms were clustered together. Revigo tree map representation of Upregulated transcripts in (d) SP_S, (e) SP_T and (f) CP condition showing GO enrichment. The treemap was built using absolute log_10_ P-value, the blocks with larger area showing categories with higher GO enrichment and the blocks with similar semantic values are represented with similar colours.

**Figure S4:** GO enrichment analysis of upregulated transcripts in CP condition [a] Biological Processes; [b] Molecular Function; [c] Cellular Components.

**Figure S5:** GO enrichment analysis of upregulated transcripts in SP_S condition [a] Biological Processes; [b] Molecular Function; [c] Cellular Components.

**Figure S6:** GO enrichment analysis of upregulated transcripts in SP_T condition [a] Biological Processes; [b] Molecular Function; [c] Cellular Components.

**Figure S7**: Correlation plot representing correlation between event specific and condition specific RT expression analysis (a) Correlation among SP_S_ss, SP_T_ss, SP_S_ov and SP_T_ov; (b) Correlation among SP_S_ss, CP_ss, SP_S_ov and CP_ov; (c) Correlation among SP_T_ss, CP_ss, SP_T_ov and CP_ov. The distribution of each variable is shown on the diagonal with bottom showing bivariate scatter plots with a fitted line top, showing the value of the correlation. The significance level is represented as stars, each significance level is associated to a symbol: p-values(0, 0.001, 0.01, 0.05, 0.1, 1) <=> symbols(“***”, “**”, “*”, “.”, " “)

**Figure S8**: Graph representing log2 fold change expression of 42 key genes using GAPDH as internal control in event specific (A) and tissue specific (B) manner. The error bar represents SD between three replicated samples.

**Supplementary Tables**

**Table S1**: Chi-square estimation of microscopy results.

**Table S2**: Summary details of assembled sequences produced after filtered reads *de novo* assembly using both CLC work bench and TRINITY.

**Table S3:** Annotation of assembled sequences (Transcripts) based on five public databases TAIR, swissprot, nr, KOG (EGGNOG) and Transcription Factors.

**Table S4:** Differential Gene Expression extracted from *de novo* assembly using EdgeR tools. Tab 1 showing DGE between SP_S and CP, Tab 2 DGE between SP_T and CP.

**Table S5:** Differential Gene Expression extracted from tea reference genome using tuxedo pipeline. The fold change was calculated between SP_S vs CP and SP_T vs CP using cuffdiff.

**Table S6:** 408 Differentially expressed transcripts encapsulating 195 genes related to fertilization and incompatibility categorized into five different phases.

**Table S7:** Gene specific interactome network analysis of differentially expressed genes.

**Table S8:** Gene specific coexpression analysis of differentially expressed genes.

**Table S9:** Primer details of selected transcripts sequences to assess relative expression analysis using quantitative Real Time-polymerase chain reaction (qRT-PCR) analysis.

**Table S10:** RNA-Seq validation of DGE with raw Ct values of reciprocal crosses and tissue specific relative expression analysis with significance test.
